# Deciphering Precise Gene Transcriptional Expression Using gwINTACT in Tomato

**DOI:** 10.3389/fpls.2022.852206

**Published:** 2022-04-14

**Authors:** Yiyang Chu, Jiachen Gong, Peiwen Wu, Ye Liu, Yinglin Du, Lili Ma, Daqi Fu, Hongliang Zhu, Guiqin Qu, Benzhong Zhu

**Affiliations:** College of Food Science and Nutritional Engineering, China Agricultural University, Beijing, China

**Keywords:** gwINTACT, nuclei purification, nuclear RNA, transcriptional expression, tomato

## Abstract

Functional gene transcription mainly occurs in the nucleus and has a significant role in plant physiology. The isolation of nuclei tagged in specific cell type (INTACT) technique provides an efficient and stable nucleus purification method to investigate the dynamic changes of nuclear gene transcriptional expression. However, the application of traditional INTACT in plants is still limited to seedlings or root cells because of severe chloroplast pollution. In this study, we proposed a newly designed and simplified INTACT based on mas-enhanced GFP (eGFP)-*Sl*WIP2 (gwINTACT) for nuclear purification in tomato (*Solanum lycopersicum*) leaves, flowers, and fruits for the first time. The yield of the nucleus purified using gwINTACT from transgenic tomato leaves was doubled compared with using a traditional INTACT procedure, accompanied by more than 95% removal of chloroplasts. Relative gene expression of ethylene-related genes with ethylene treatment was reevaluated in gwINTACT leaves to reveal more different results from the traditional gene expression assay based on total RNA. Therefore, establishing the gwINTACT system in this study facilitates the precise deciphering of the transcriptional status in various tomato tissues, which lays the foundation for the further experimental study of nucleus-related molecular regulation on fruit ripening, such as ChIP-seq and ATAC-seq.

## Introduction

The critical status of transcriptional regulation on gene expression has been known for more than half a century (Jacob and Monod, 1961). Studies on the transcriptional control of functional gene expression are mainly based on total RNA or total mRNAs, bringing about two potential issues. The nucleus is crucial for gene transcriptional expression to regulate plant physiological activities (Fazal et al., 2019), and pre-mRNA production and modification are also performed in the nucleus (Lau et al., 1991; Nunez et al., 2009). However, the nuclear RNA accounts for only 10%–20% of the total RNA (Piwnicka et al., 1982). In addition, mRNAs of total RNA have different half-lives and may degrade during transportation from the nucleus to the cytoplasm, making it difficult to detect the nascent transcription or the changes of undergoing translation strictly by these steady-state transcripts (Park et al., 2012). Therefore, compared with total RNA, RNAs from the nucleus are supposed to predict the real-time expression level of genes more accurately, and obtaining high-quality nuclear RNA by extracting it from the purified nucleus becomes a critical step for studying functional gene regulation.

Currently, there are many methods for nuclei isolation and purification, including differential centrifugation, fluorescence-activated nuclei sorting (FANS; Zhang et al., 2008), and isolation of nuclei tagged in specific cell types (INTACTs; Deal and Henikoff, 2010, 2011). INTACT is one of the most promising methods for nuclear purification; it was first described in *Arabidopsis* and later extended to *Caenorhabditis elegans* (Deal and Henikoff, 2010; Steiner et al., 2012). This approach labels the nucleus with an encoded tag, and after the marked nuclei are purified, the transcriptional profiles can be illustrated by the nuclear RNAs (Henry et al., 2012). Compared with other approaches, the INTACT procedure does not rely on other sophisticated equipment or specialized skills, and the affinity-purification process can be easily performed (Deal and Henikoff, 2010, 2011). There are two fundamental functional units for this technology. One is a nuclear tagging fusion (NTF) protein composed of a biotin ligase target peptide (BLRP) for biotinylated nuclei, a green fluorescent protein (GFP) for visualization, and an outer nuclear membrane targeting domain (WPP) for accurate targeting on the nuclear envelope (Del Toro-De Leon and Kohler, 2019). The other component is an *Escherichia coli* biotin ligase (BirA) that is co-expressed with the NTF protein in plants for binding with streptavidin-coated magnetic beads to remove the contamination in crude-extracted nuclei (Deal and Henikoff, 2011; Henry et al., 2012; Steiner and Henikoff, 2015).

The INTACT has been applied in *Arabidopsis*, rice, and tomatoes (Deal and Henikoff, 2010; Ron et al., 2014; Reynoso et al., 2018) to investigate the transcriptional expression of genes in plants. In addition, there are numerous optimizations of vectors and purification equipment used in INTACT. Streptavidin-coupled magnetic beads were replaced with GFP antibody-coupled magnetic beads to improve the nuclear purity of *Arabidopsis* embryo cells to 70–90% (Park et al., 2016). Magnetic frame was substituted for the original flow adsorption device to shorten the adsorption time of magnetic beads (Wang and Deal, 2015). In *Oryza*, the WPP in NTF was superseded with the WPP-interacting protein (WIP) with a more vital binding ability (Rose and Meier, 2001; Xu et al., 2007; Zhao et al., 2008; Reynoso et al., 2018). However, despite many improvements in INTACT, its application is still limited to seedlings or root tissues. In addition, it has not been used in leaves because of the severe chloroplast contamination or fruit tissues where nuclei are few and challenging to extract.

This study established a precise nuclear regulation research system, namely gwINTACT [INTACT based on mas-enhanced GFP (eGFP)-*Sl*WIP2], in different tissues of tomato (*S. lycopersicum*), a model material for studying plant growth and development, especially fruit ripening (Giovannoni, 2007; Osorio et al., 2020). The removal of the chloroplasts and the yield of the target nucleus were enhanced compared with the conventional INTACT. The ethylene regulation on genes related to the self-synthesis and signal transduction pathway was precisely deciphered. This study has provided access for characterizing the accurate gene regulatory networks of different tomato tissues, from growth and development to fruit ripening.

## Materials and Methods

### Plant Material and Growth Conditions

Tobacco (*Nicotiana benthamiana*) and tomato (*S. lycopersicum*, Micro-Tom) seedlings were cultured in a growth chamber with 16 h light and 8 h dark cycles at a temperature of 25°C. Plant tissues were harvested, frozen immediately in liquid nitrogen, and stored at −80°C for further RNA studies.

### Nuclear Tagging Fusion Plasmid Construction

The *SlWIP1* (*Solyc10g005720*), *SlWIP2* (*Solyc07g066170*), mannopine synthase promoter (*pmas*), *eGFP*, *BLRP*, and *BirA* were cloned according to the sequence in the NCBI^1^ and previous INTACT research (Reynoso et al., 2018). All primers used for construction are listed (Supplementary Table 1A). The eGFP-*Sl*WIP2 (GW) was fused by overlap PCR, and a linker peptide with 7aa (S-G-A-A-A-A-A) was added between the two proteins to avoid the interactions.

The GW was introduced into the pCAMBIA1300-221 vector *via* digestion (*Bam*HI/*Sac*I) and T4 ligation to generate a pCAMBIA-35S-GW fusion plasmid, called GW1. *Mas* was inserted into the GW1 and substituted for the CaMV 35S promoter (*p35S*) to form pCAMBIA-mas-GW plasmid by digestion (*Pme*I/*Xba*I) and ligation, called GW2. The BLRP was fused with GW through overlap PCR. The fusion protein was ligated by *Bam*HI/*Sac*I, and *BirA* was ligated using a unique *Eco*RI site to generate a pCAMBIA-35S-BirA-nos-mas-BLRP-GW recombinant vector, called BGW.

The localization prediction of *Sl*WIP1 and *Sl*WIP2 was performed using the bioinformatic deep learning method.^2^ Subcellular localization of GW2 and BGW was predicted by CELLO2GO.^3^

### Tobacco Transient Expression and Tomato Genetic Transformation

The *Agrobacterium tumefaciens* strain GV3101 was used to express the *Sl*NTF constructs transiently in *N. benthamiana* (Luo et al., 2017). The tobacco protoplast was isolated by referring to the method provided by the Institute of Botany, Chinese Academy of Sciences (Lei et al., 2015).

The GW2 and BGW *Sl*NTFs were transformed into tomatoes using the same *A. tumefaciens* strain, GV3101, as described in our previous studies (Li et al., 2018). The positively transformed lines were identified by screening for hygromycin resistance. At least three lines were obtained for each assay. T_0_ and T_1_ lines of transgenic tomatoes were used for further analyses.

### Protein Isolation and Western Blotting

Proteins from leaf and fruit tissues were extracted using a previously described protocol (Wang et al., 2006). Samples (0.5 g) were ground into powder in the liquid nitrogen and transferred to 2-ml centrifuge tubes. Trichloroacetic acid (TCA) (10%) was added to the samples, mixed well, and centrifuged at 4°C. The supernatant was removed, and ammonium acetate solution (0.1 M) was added. After mixture and centrifugation, the supernatant was discarded, and the sediment was mixed with 80% acetone and centrifuged. The supernatant was removed again, and samples were air-dried for 20 min. Tris-phenol (pH 8.0) and SDS solutions (30% sucrose, 2% SDS, 5% β-mercaptoethanol, 0.2 M Tris, and pH 8.0) were added to isolate proteins. The crude proteins were precipitated with ammonium acetate (0.1 M) by staying overnight at −20°C. Then, the pellets were washed with 80% acetone and 100% methanol. After being clarified by centrifugation, the sediment was finally dissolved in SDS buffer (0.5 M Tris, pH 7, and 1.4% SDS).

Protein extracts were separated on 12% (w/v) SDS-PAGE gels and used in immunoblot analyses. Proteins were transferred to a polyvinylidene fluoride (PVDF) membrane and blocked in 5% non-fat milk overnight at 4°C. Rabbit polyclonal monoclonal antibody was added at a ratio of 1:1,000 and incubated overnight at 4°C. The membranes were washed with Tris-buffered saline plus Tween 20 (TBST) three times, 8 min each time. GW2 and BGW were then detected using an anti-eGFP antibody (1:2,000; Solarbio, Beijing, China) and incubated for 2 h at room temperature. After three washes with TBST, the PVDF membrane was visualized using a chemiluminescence reagent (Luminata Crescendo Western HRP Substrate; Millipore, MA, United States).

### Laser Confocal Microscopy and Phenotype Analysis

All primers used for construction are listed (Supplementary Table 1A). The *eGFP* gene was cloned and introduced into pCAMBIA1300-221 plasmid *via* digestion (*Bam*HI/*Sac*I) and T4 ligation to generate pCAMBIA-35S-eGFP plasmid. *Mas* was inserted into the pCAMBIA-35S-eGFP by digestion (*Xba*I/*Pme*I) to form pCAMBIA-mas-eGFP plasmid. The two recombinant vectors, pCAMBIA-35S-eGFP and pCAMBIA-mas-eGFP, were constructed as a negative control for subcellular localization of the outer nuclear membrane. Subcellular localization and fluorescence detection of tobacco and tomatoes expressing different *Sl*NTF constructs were performed using a laser confocal (#A1RMPSi, Olympus, Tokyo, Japan). Phenotype images of GW2 and BGW transgenic tomato lines were obtained using a camera (#EOS 750D, Canon, Tokyo, Japan).

### Biotin Content Measurement and Ethylene Treatment

The biotin content of leaf tissues from *GW2* overexpressing (*GW2*-OE) lines and *BGW*-OE lines was measured by a Biotin Quantitative Detection Kit (Elabscience, Wuhan, China) according to the manufacturer’s instructions. The entire tomato lines of 21-day-old seedlings expressing GW2 were put in an airtight chamber fitted with inlet and outlet ports (Li and Guo, 2018). The chamber was connected to an air flow-through system that contained ethylene at 100 μl/L, at a rate of 100 ml/min. The control group was treated with dry air substituted for ethylene in the same condition. The treatment was conducted for 4 h at 25°C in the greenhouse. After the treatment, leaves (0.1 g) were frozen in liquid nitrogen and used for total RNA isolation. In addition, leaves (0.3 g) were collected and rapidly transferred to a petri dish on ice for nuclear RNA extraction. Three biological replicates were included for each treatment, and each replicate was obtained from independent sampling.

### Nuclear Isolation and Purification

Nuclei purification buffer (NPB) was prepared as 20 mM 4-propanesulfonyl morpholine (MOPS), pH 7.0; 90 mM potassium chloride (KCl), 40 mM sodium chloride (NaCl), 2 mM ethylene diamine tetraacetic acid (EDTA), 0.5 mM Ethylene glycol bis(2- aminoethyl ether)-N,N,N’,N’- tetraacetic acid (EGTA), 0.5 mM spermidine, and 0.2 mM spermine. Nuclei were isolated from fresh tissue, as described previously (Deal and Henikoff, 2011), with only a few modifications. For the test of target nuclei yield, leaf tissues (0.5 g), flower tissues (0.5 g), or fruits (1 g) were shredded with a double-sided blade in 10 ml of ice-cold NPB containing 1 × complete EDTA-free protease inhibitor cocktail (Roche, Basel, Switzerland). For the detection of more visible fluorescence changes, leaf tissues (1 g), flower tissues (1 g), or fruits (5 g) were used, and the treatment was the same as the test of target nuclei yield. The homogenized extracts were filtered through a 40-μm nylon mesh (fruit tissue was filtered twice, the first time with one layer of filter cloth, and the second time with two layers of cloth), and centrifuged at 1,500 g for 15 min at 4°C to enrich the nuclei. The supernatant was removed, and nuclei were resuspended in 1-ml NPB buffer. These crude extracted nuclei were used for affinity purification.

For the nuclei isolated from *BGW*-OE lines, the traditional INTACT procedure based on streptavidin beads (Reynoso et al., 2018) was used for purification. Notably, 25 μl of streptavidin M280 Dynabeads (Invitrogen, CA, United States) were washed with 1-ml NPB, resuspended to 25 μl, and added to 1 ml crude nuclei extract. The mixture was gently rotated at 4°C for 30 min, diluted to 14 ml with NPB mixed with 0.1% (v/v) Triton X-100 (NPBt), transferred to a 15-ml tube, and placed in a 15 ml magnetic frame for 2–7 min at 4°C. The supernatant was carefully removed, and the beads were diluted to 14 ml again (this wash step was repeated twice). Bead-bound nuclei were resuspended in 1 ml of NPBt and transferred to a new 1.5-ml tube. For the nuclei separated from *GW2*-OE lines, gwINTACT relying on eGFP-bead capture was performed, as described previously (Henry et al., 2012). Of note, 12 μl of Pierce Protein A/G Magnetic Beads (Thermo Fisher Scientific, MA, United States) were washed with NPB and resuspended to 400 μl, added to 8 μl of anti-eGFP polyclonal antibody (Solarbio), and incubated for 30 min at 4°C. The eGFP-beads were added to the enriched crude nuclei and incubated for 15 min. The mixture was then diluted to 12 ml with NPBt, and beads were collected on a magnet and washed three times with 12 ml NPBt for 2 min each time. The beads-nuclei mixture was resuspended using 400 μl of NPBt and transferred to a new 1.5-ml tube. Of note, 10 μl of the beads-nuclei mixture was stained with 1 × 4′,6-diamidino-2-phenylindole (DAPI) for fluorescence observation and counting. For RNA and DNA extraction, the nuclei were held using a 2-ml tube magnet and resuspended in 20 ml NPB before performing the extraction.

### Extraction of Nuclear DNA, Total and Nuclear RNA, and RT-qPCR

The nuclear DNA from different tomato tissues was extracted using M5 Microclinical Genomic DNA Rapid Extraction Kit (Mei5bio, Beijing, China). Nuclear RNA from *GW2* transgenic tomato lines was isolated using GenElute Single-Cell RNA Purification Kit (Sigma-Aldrich, MO, United States). Total RNA was extracted using the E.Z.N.A. Plant RNA kit (OMEGA, GA, United States). RNA quality and quantity were verified by 1.5% (v/v) agar gel electrophoresis and microspectrophotometer (Thermo Fisher Scientific). Then, cDNA was synthesized using M-MLV reverse transcriptase (Promega, WI, United States). The Micro-Tom *ACTIN* gene (*Solyc03g078400*) was used as an internal reference. All gene-specific primers were designed (Supplementary Table 1B), and RT-qPCR was conducted using TransStart Top Green qPCR SuperMix (TransGen, Beijing, China). The values of relative gene expression were calculated using the 2^–ΔΔCt^ method (Livak and Schmittgen, 2001). Three biological replicates from independent sampling were included for each point.

### RNA-seq and Bioinformatics Assay

The tomato leaves from *BGW*-OE lines, *GW2*-OE lines, and WT control group were collected for RNA-seq to analyze the biotin anabolism. The *GW2*-OE tomato leaves with ethylene treatment and control group (treated with dry air) were monitored using RNA-seq to decipher the ethylene regulation. Three biological replicates from independent sampling were included for each point. At least 6 GB of raw data per sample were generated and pretreated from the pair-end sequencing performed on Illumina Novaseq6000 PE150 by Novogene. Clean reads were checked for quality using the threshold Q < 20 and mapped to the tomato reference genomes (ITAG2.4) using TopHat2 (version 2.0.8). Unique alignments with no more than 2 nucleotide mismatches were used to construct transcripts using Cufflinks (version 2.0.2). Differentially expressed genes (DEGs) were confirmed with the selection criteria of | log_2_^fold change^| ≥ 1, *p*-adjust < 0.05. Then, KEGG enrichment analysis of DEGs was performed using KOBAS (version 2.0), based on organism annotation libraries and native BLAST tools. KEGG pathways with *p* < 0.05 for each sample were visualized using R Project (version 3.4.0). The clean data of these RNA-seq for this study were submitted to the NCBI Sequence Read Archive under accession number PRJNA795585.

### Statistical Analysis

SPSS (SPSS Statistics, version 22.0) and Microsoft Excel 2021 were used for statistical analyses to conduct one-way ANOVA analysis, and statistical significance was set at *p < 0.05 and ^**^p < 0.01.

## Results

### *Sl*NTFs Design and Vector Construction

**FIGURE 1:**
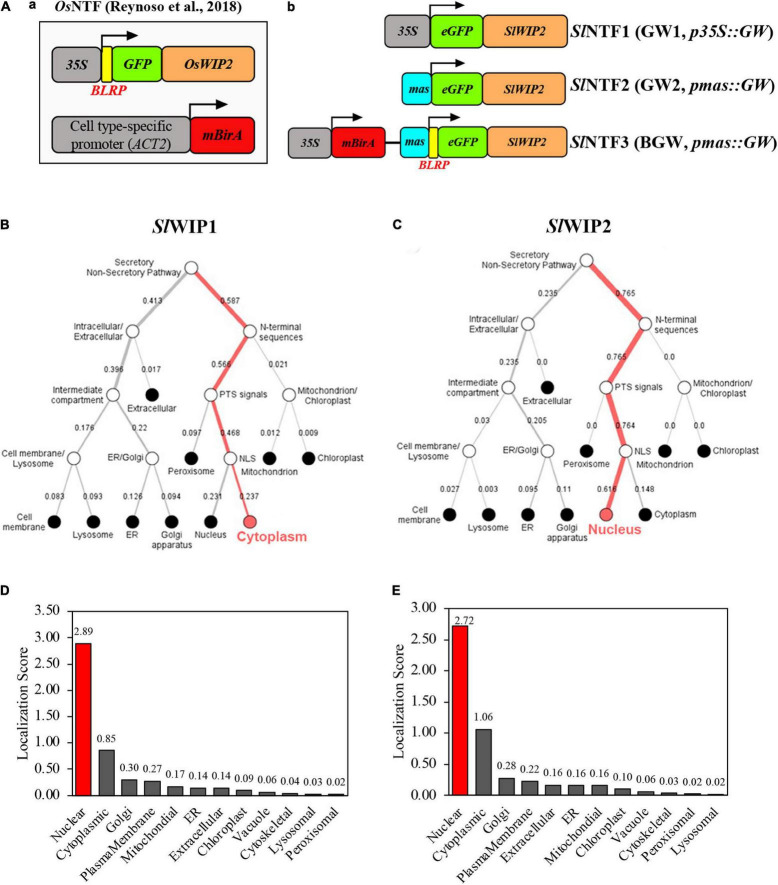
Diagram of three different *Sl*NTF proteins and their localization prediction. **(A)** Three modified nuclear tagging fusion (NTF) proteins were used in this study. Conventional NTF (left) designed in *Oryza sativa* L. (*Os*NTF) was consisted of three fundamental units: a C-terminal region of *Os*WIP2 protein for targeting to the surface of the nuclear membrane, a GFP for observation, and a biotin ligase target peptide along with BirA system for biotinylation of the nuclear envelope. Three retooled NTF proteins were shown (right), namely, *p35S:eGFP-SlWIP2* (GW1), *pmas:eGFP-SlWIP2* (GW2), and BGW, which was composed of BirA system and GW2 in a common vector. **(B,C)** Localization prediction of different WPP-interacting protein (WIP) proteins in *S. lycopersicum* using bioinformatic deep learning methods (https://services.healthtech.dtu.dk/service.php?DeepLoc-1.0). **(D,E)** Subcellular localization prediction of GW2 and BGW with CELLO2GO (http://cello.life.nctu.edu.tw/cello2go/).

Aiming to provide an INTACT procedure for various tomato tissues that satisfied the nucleus purification effect, we integrated multiple modifications of INTACT based on previous studies (Figure 1A-a). First, the biotinylation system was replaced with eGFP to improve the target nucleus yield, and the redesigned INTACT with GW does not rely on the streptavidin-mediated capture of biotinylated nuclei (Henry et al., 2012; Steiner and Henikoff, 2015; Agrawal et al., 2019), facilitating the construction and procedure (Figure 1A-b).

Second, WIP was substituted for WPP to anchor the outer nuclear membrane as its better performance on nuclear envelope location was proven in the study of *Arabidopsis* (Reynoso et al., 2018). There are two tomato orthologs of *OsWIP2* (LOC_Os09g30350.1) in rice and *WIP1* (*At4g26450*) in *Arabidopsis*, including *SlWIP1* (*Solyc10g005720*) and *SlWIP2* (*Solyc07g066170*). The localization prediction of the two *Sl*WIP proteins shows that *Sl*WIP1 is located in the plasma membrane, while *Sl*WIP2 is in the outer nuclear envelope (Figures 1B,C). As a result, *SlWIP2* was applied in the subsequent research. Moreover, to circumvent the duplicate promoter in vector and guarantee the non-tissue-specific expression in tomatoes, different promoters were used to drive the *Sl*NTFs. Mas promoter (*pmas*) was proven to enhance the editing events in the previous study of poplar 84K by driving Cas9 (An et al., 2021). Accordingly, we hypothesized that the *pmas* could increase the direct expression of *Sl*NTFs in tomatoes compared with the commonly used *p35S*. Therefore, three distinct *Sl*NTFs were designed to evaluate nuclear envelope localization and target nuclear purification (Figure 1A-b). For *Sl*NTF1 driven by *p35S* (35S-GW, GW1) and *Sl*NTF2 driven by *pmas* (mas-GW, GW2), GW was used to take the place of the biotinylation system. *Sl*NTF3 was designed as a control group consisting of the similar units as the traditional NTF (Deal and Henikoff, 2010), *p35S:BirA*, and *pmas:GW*. Compared with the previous NTF, the two components of *Sl*NTF3 were not separated and constructed in a common standard vector (35S-BirA-nos-mas-BLRP-GW, BGW). Finally, the outer nuclear membrane localization of different *Sl*NTFs was confirmed by subcellular localization prediction with CELLO2GO (Figures 1D,E).

### Nuclear Envelope Location of *Sl*NTFs in Tobacco

**FIGURE 2:**
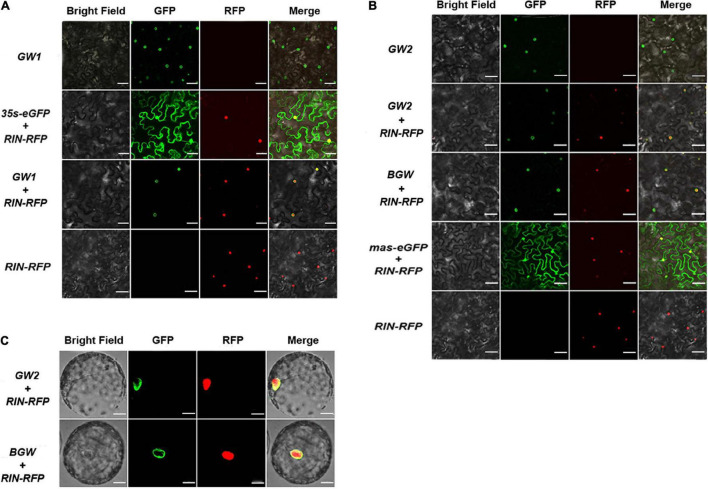
Subcellular localization of three *Sl*NTFs in tobacco. Different *Sl*NTF proteins were transiently overexpressed in tobacco leaves **(A,B)** and protoplast **(C)**. *p35S:RIN-RFP* was used as a positive control for nuclear localization, and a negative control (NC) was designed with *p35S:eGFP* and *pmas:eGFP*. Scale bars: A and B, 100 μm; C, 20 μm.

Transiently overexpressed tobacco was produced, expressing each *Sl*NTF version to verify preliminary nuclear membrane localization. Confocal microscopy was applied to detect the cellular distribution of the three *Sl*NTF proteins in leaf cells and protoplasts. The *p35S:eGFP* and *pmas:eGFP* were used as a negative control with their widespread fluorescence signal position. Although *RIN* is a critical transcription factor for fruit ripening regulation and is accurately located in the nucleus (Li et al., 2018), *p35S:RIN-RFP* was used to indicate the nuclear localization with red fluorescence signal. Meanwhile, the *GW1*, *GW2*, and *BGW* were co-expressed, respectively, with *p35S:RIN-RFP.* An apparent green circle formed on the nuclear surface, which was filled with the red fluorescence (Figures 2A,B). The two negative controls were also expressed with *p35S:RIN-RFP*, and relatively, the GFP signal emerged in the nucleus and cytoplasm with no separate ring around the nuclear envelope, demonstrating that the three *Sl*NTFs are closely associated with the nuclear envelope. In addition, there is no detectable difference between GW1, GW2, and BGW in the subcellular distribution of tobacco cells, indicating that *Sl*NTFs driven by different promoters performed equally well on the nuclear envelope localization. To ensure the *Sl*NTF constructs could be used in other studies of functional genes which commonly promoted by the *p35S*, GW2 and BGW driven by *pmas* were chosen for the subsequent research.

**FIGURE 3:**
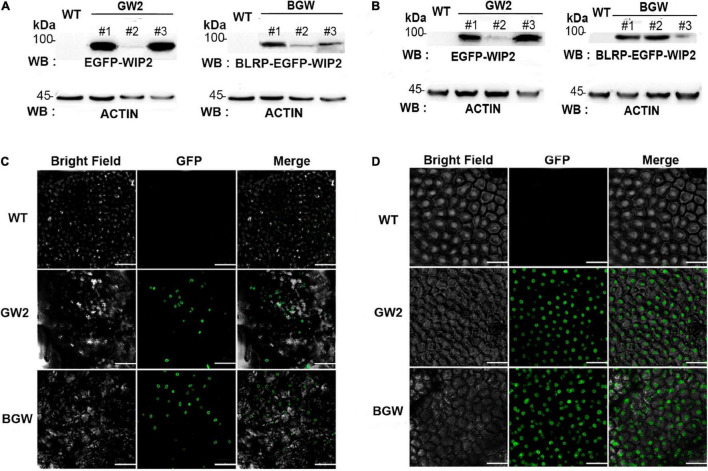
Detection of three *Sl*NTF proteins in different tomato tissues. Western blotting (WB) was performed with anti-eGFP polyclonal antibody in leaves **(A)** and fruits **(B)**, respectively. ACTIN protein is used as a quantitative control. Microscopy images of GFP fluorescence in leaves **(C)** and fruits **(D)** showed that both GW2 and BGW are located in the outer nuclear membrane. Scale bars: C, 50 μm; D, 100 μm.

Protoplasts of tobacco were collected by enzymatic hydrolysis to confirm the nuclear localization of GW2 and BGW (Figure 3C). The green fluorescence was observed on the nuclear surface in both GW2 and BGW, further verifying that GW2 and BGW could locate on the outer nuclear membrane.

### Detection of *Sl*NTFs by Nuclear Membrane Anchored With eGFP in Tomatoes

Independent transgenic tomato lines were obtained that overexpress *Sl*NTFs with stable inheritance under the control of the *pmas* (eight GW2 lines and seven BGW lines) *via Agrobacterium* transformation. The positively transformed lines were identified using hygromycin resistance screening. The relative expression of *SlWIP2* and *BirA* was evaluated by RT-qPCR.

Western blotting (WB) with anti-enhanced GFP (eGFP) polyclonal antibody was performed to monitor the GW protein (92 kDa) in GW2 and BLRP-GW protein (94.9 kDa) in BGW in the different tomato tissues. Strong protein signal was detected in both leaf and fruit tissues from the above two *Sl*NTFs expressing lines (Figures 3A,B). Specifically, line1 and line3 of GW2 had a significantly higher expression level of protein than other groups, which was consistent with the RT-qPCR results (Supplementary Figure 1). Moreover, to verify the outer nuclear membrane location in GW2 and BGW tomato lines, subcellular localization was performed in leaf tissues and fruits to confirm the fluorescence distribution (Figures 3C,D). The strong halo of GFP signal surrounding the nuclei in the two types of *Sl*NTF-expressing plants indicates the integral association of *Sl*WIP2 with the outer nuclear membrane.

These results demonstrate that the chimeric *Sl*NTF driven by *mas* promoter (GW2 and BGW) can anchor the outer nuclear membrane in different tomato tissues, supporting the application of affinity purification of eGFP-tagged nuclei in tomatoes.

### Enhanced Effect on Nuclear Purification by gwINTACT

**FIGURE 4:**
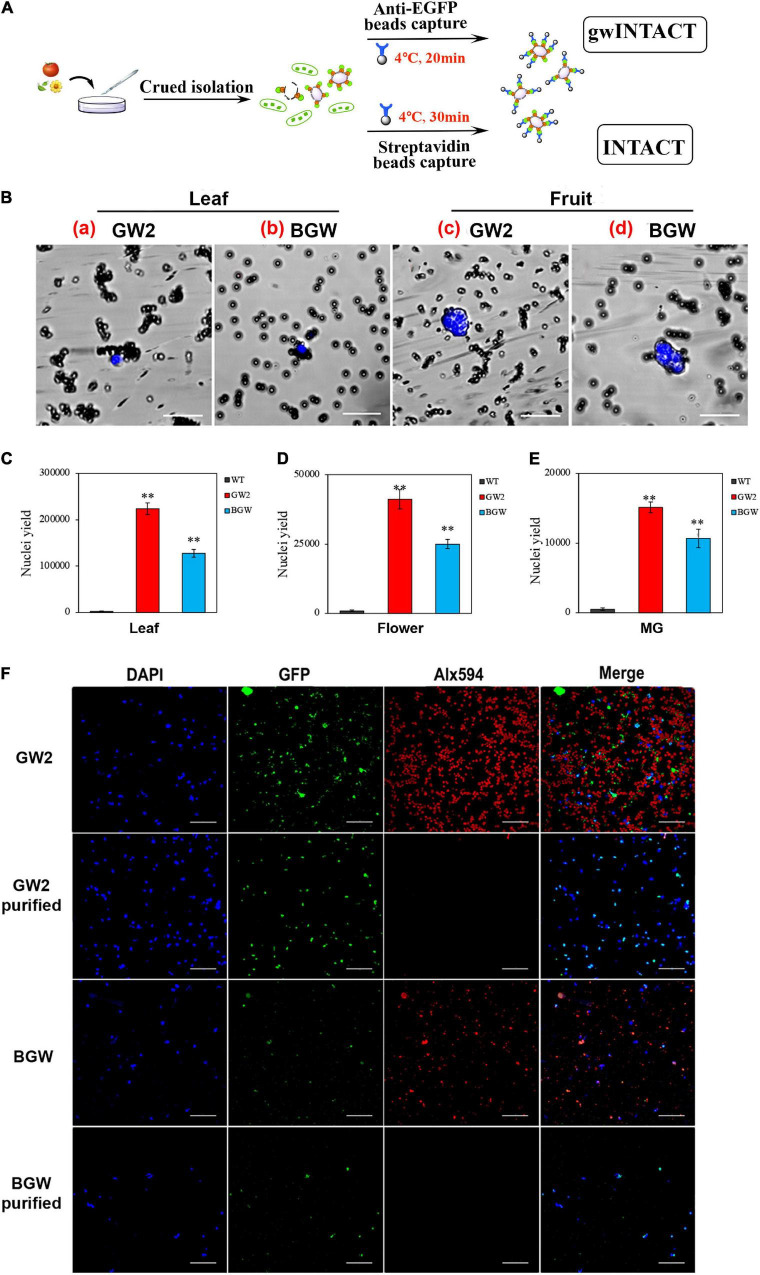
Nuclei separation using INTACT with GW2 protein. **(A)** The workflow diagram of two different INTACT strategies. The INTACT procedure with GW2 (gwINTACT) is different from BGW and does not rely on the streptavidin-mediated capture of biotinylated nuclei. **(B)** Microscopy images of purified bead-binding nuclei from different tomato tissues of GW2 lines or BGW lines. Nuclei were stained with 4′,6-diamidino-2-phenylindole (DAPI; blue) and surrounded by magnetic beads (dark gray) coated with streptavidin (BGW) or eGFP (GW2) antibody. a and b are from leaf tissues, and c and d are from fruit tissues. A single-tagged-nucleus can be bound to several beads. **(C-E)** Yield of purified nuclei from leaf, flower, and MG fruit tissues expressing GW2 or BGW. The purified nuclei were counted using a hemocytometer. **(F)** Fluorescence images of nuclei and chloroplasts from leaf tissues of GW2 lines and BGW lines with purification. Nuclei were visualized with DAPI (blue) and GFP (green), and chloroplasts were observed by Alx594 (red). Scale bars: B, 20 μm; F, 50 μm. Asterisks indicate significant differences (^**^p < 0.01) in comparison with wild type (WT).

Two INTACT procedures were established, respectively, based on GW2 (gwINTACT) and BGW (conventional INTACT) in leaves, flowers, and fruit tissues of transgenic tomato lines. In addition, the gwINTACT strategy was shown to have superior nuclear yield performance and reduce organelle pollution. The work scheme of the two INTACT strategies is shown (Figure 4A), the labeled nuclear membrane protein could combine with the affinity beads, and the target nucleus was grasped through magnetic adsorption. Compared with the traditional INTACT, which incubated the nucleus with beads at 4°C for 30 min (Deal and Henikoff, 2011), the gwINTACT could be operated for 15–20 min, time-saving but not impairing the purification. Moreover, the captured nucleus was stained by DAPI to monitor by fluorescence microscope observation (Figure 4B). The black magnetic beads wrapped around the blue nucleus can be seen under the laser confocal microscope, indicating the tight integration between affinity beads and the nucleus in *GW2*-OE and *BGW*-OE lines. Therefore, two INTACT procedures relied on distinct affinity magnetic beads were obtained for nuclear purification in various tomato tissues.

Because the tissue-specific nuclei of leaves, flowers, and fruits from tomatoes expressing the two *Sl*NTF versions were separated and purified by using the two corresponding INTACT protocols. We compared the target nuclear yield and the purification effect. The pure nuclei were subsequently diluted with NPB buffer, and the counts of intact nuclei stained with DAPI were calculated precisely using the blood cell counting plate (Figures 4C–E). The number of nuclei purified from 0.5 g of GW2 leaves can yield 2.2 × 10^5^ target nuclei, double that of BGW. For GW2 lines, 4.1 × 10^4^ and 1.5 × 10^4^ pure nuclei were obtained from 0.5 g of flower tissues and 1 g of fruit tissues, respectively, which were not reported in previous studies and were nearly twice that of BGW. These results illustrate that gwINTACT could be employed in various tomato tissues and could allow for a more intensive nuclear yield.

The nucleus was covered with green fluorescence of eGFP and stained with DAPI, and the chloroplast had spontaneous red fluorescence. Therefore, changes in the quantity of the nuclei and chloroplast with purification were detected through the fluorescence signal observation (Figure 4F and Supplementary Figure 2). The depuration consequence of the two INTACT procedures based on the different *Sl*NTFs was evaluated by fluorescence variation. Almost all of the red signals of Alx594 disappeared after purification, demonstrating that both of the two strategies have a visible effect on chloroplast elimination. Moreover, fewer nuclei remained after bead adsorption in BGW, while numerous intact nuclei were drafted *via* gwINTACT. We surmised that gwINTACT could prevent the nucleus from being damaged during the purification procedure. To further confirm the relative content of residual chloroplast genes in the purified product, PCR was performed to probe the nuclear and chloroplast-specific genes after bead pull-down (Supplementary Figure 3). For the leaf cells that experienced severe chloroplast contamination, the chloroplast removal rates were more than 95% in GW2 lines, while the chloroplast removal rates were 84% in BGW lines. In addition, in all tomato tissues, namely, leaves, flowers, and fruits, the relative content of chloroplast genes in GW2 lines was more visibly decreased after purification than that in BGW, further indicating the enhanced purification performance with gwINTACT.

### Analysis of Phenotype and Biotin Anabolism to Transgenic Tomato Lines

**FIGURE 5:**
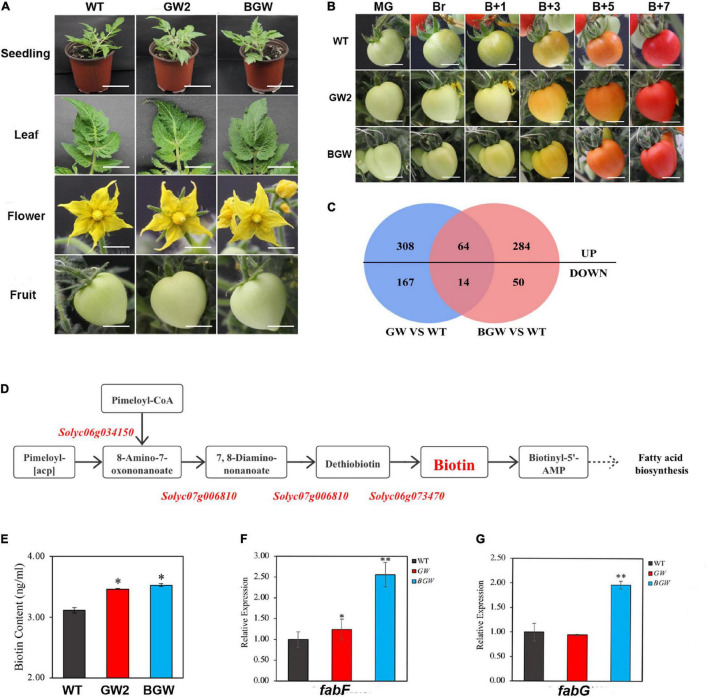
Phenotype and transcriptomic analysis in GW2 lines and BGW lines. **(A,B)** Phenotype comparison among GW2, BGW, and WT lines during growth and development and fruit ripening. A, Different tissues were shown, including 14-day-old seedlings, leaves, flowers in full bloom, and green fruit. B, Fruit ripening stage of WT, GW2, and BGW lines at different time points. (mature green, MG; breaker, Br; breaker + 1 days, B + 1; breaker + 3 days, B + 3; breaker + 5 days, B + 5; breaker + 7 days, B7). **(C)** Venn diagrams showing the statistical counts of differentially expressed genes identified in GW2 and BGW compared with WT. **(D)** Diagram of the biotin synthesis pathway in tomato, all three critical genes were marked in red. **(E)** Results of biotin content measured in leaves from WT, GW, and BGW lines. Relative gene expression in biotin-related downstream pathways, such as fatty acid biosynthesis **(F,G)**, was measured in leaves from GW2 lines or BGW lines. Scale bars: A, Seedling, 5 cm. A, Leaf, flower, and fruit, and B, 1 cm. Asterisks indicate significant differences (*p < 0.05, ^**^p < 0.01) in comparison with WT.

To monitor the impact of expressing *Sl*NTFs, we detected the phenotypes of the transgenic tomato materials and genes involved in the biotin-related pathway. Compared with the wild-type, there was no apparent discrepancy between *GW2*-OE lines and *BGW*-OE lines, such as plant height and architecture, flower colors and shape, fruits shape, and ripening (Figures 5A,B and Supplementary Figure 4), indicating the undetectable phenotypic consequence of GW2 and BGW expression on the development, fertility, or fruit ripening under standard greenhouse conditions.

The DEGs in leaf transcripts from the tomato expressing GW2 or BGW were authenticated in comparison with the WT by RNA-seq. Five hundred and fifty-three DEGs were identified in GW2, while 412 DEGs were identified in BGW (Figure 5C). Notably, there were no significant differential expressions of the three critical biotin-synthesis genes (Entcheva et al., 2002; Figure 5D; Supplementary Table 2) and no significant changes in the biotin content of the two *Sl*NTF-overexpressing lines (Figure 5E). This indicates that both *Sl*NTF proteins have less influence on biotin synthesis in tomato plants. In addition, biotin is a vital cofactor of many important carboxylases/decarboxylases (Knowles, 1989; Jitrapakdee and Wallace, 2003; Lin and Cronan, 2011). The biotin precursor pimeloyl-acyl carrier protein (ACP) was proved to originate from the fatty acid biosynthesis pathway in a previous study of *Corynebacterium glutamicum* (Ikeda et al., 2017). Combined with the RT-qPCR and RNA-seq results, two crucial genes, namely, *Solyc03g122120* (*fabF*) and *Solyc06g071070* (*fabG*), involved in both biotin metabolization and fatty acid synthesis, were found to change distinctly in BGW lines with upregulation by 2.5- and 2-fold, respectively (Figures 5F,G). However, there was no differential expression of the two genes in GW2 lines. We speculated that the impact of the biotin-related pathway was alleviated by the removal of the *BLRP* and *BirA* (Peters-Wendisch et al., 2012; Ikeda et al., 2017).

These results support the finding that both GW2 and BGW had no profound affection on plant growth and development and the synthesis of endogenous biotin. However, for some biotin-related pathways, GW2 may have less influence on plants. Therefore, a convenient and efficient gwINTACT procedure has been established for nuclear purification with universal usability in various tomato tissues to investigate the regulation of gene transcriptional expression.

### Precise Expression of the Ethylene-Relative Genes

**FIGURE 6:**
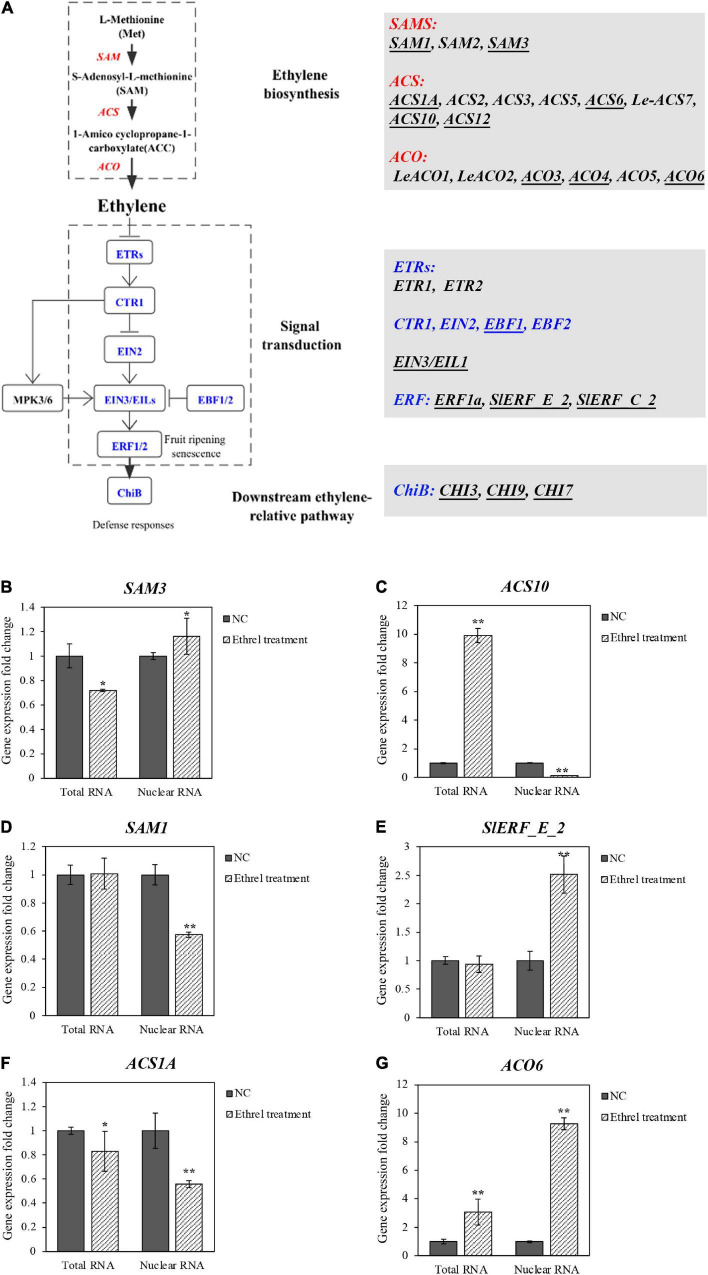
Relative expression of ethylene-related genes in total RNA and nuclear RNA from leaves of GW2 lines with ethylene treatment. **(A)** Sketch map of ethylene synthesis and signal transduction pathways; all genes in these pathways were listed. Essential genes with relatively higher expression value (RPKM; Tomato Genome, 2012) in tomato leaves were underlined, which is shown as reverse changes **(B,C),** different changes **(D,E),** alike **(F,G)** in comparison with total RNA and nuclear RNA. Group of GW2 without ethephon treatment is used as a NC. Asterisks indicate significant differences (*p < 0.05, **p < 0.01) in comparison with WT.

Ethylene is essential for regulating plant growth, development, and fruit ripening through the upstream synthetic pathway and downstream signal transduction pathway (Wang et al., 2017). For a precise illustration of gene transcriptional expression, we took the research on the regulation of ethylene on genes related to the self-synthesis and signal transduction pathway as an example, and 30 essential genes were listed (Figure 6A). The obtained high-efficiency gwINTACT was applied in GW2 transgenic tomato material for pure nucleus and superior nuclear RNA.

First, tomato leaves from *GW2*-OE lines were treated with ethylene, and 4,891 DEGs preliminarily identified through transcriptome analysis of the total RNA by RNA-seq (Supplementary Figures 5A,B). Ten crucial genes involved in the ethylene synthetic pathway and signal transduction pathway were identified by KEGG (Figure 6A and Supplementary Tables 3A–C). Subsequently, the other 20 genes in ethylene synthesis and signal transduction pathways were filtrated, according to the reads per kilobase per million mapped reads (RPKM) of tomato leaves. Genes that had an RPKM value of less than 3 were removed. As a result, 17 genes were selected specifically for the study of ethylene regulation (Supplementary Figure 6 and Supplementary Table 3D). The nucleus from the ethylene-treated and untreated GW2 tomato leaves was extracted and purified by gwINTACT, and RT-qPCR was conducted to detect the different relative gene expression levels between nuclear and total RNA. Finally, we identified 14 DEGs by total RNA, while 16 DEGs were obtained from the nuclear RNA.

Specifically, eight genes have different expression fold change levels compared with the total RNA, of which four genes showed even opposite change levels (Supplementary Figures 6B,D,E,O). *SAM3*, the crucial synthetase used to generate SAM in the synthesis pathway, was monitored with downregulation from total RNA, while the nuclear RNA demonstrated an upregulation (Figure 6B). The same contradictory situation was found for *ACS10* (Figure 6C). In addition, the other four genes showed undetectable changes in gene expression from one kind of RNA preparation but a significant variation from the other method. Among them, the gene expression change levels of *SAM1*, *EIN3/EIL1*, and *SlERF_E_2* are failed to be detected by total RNA, whereas they are detected by nuclear RNA differently with 0.5-fold reduction and 1.5-fold upregulation, respectively (Figures 6D,E; Supplementary Figure 6K). As for the *ACO4*, the change of the gene expression level detected by total RNA was significant, but not obvious when detected by nuclear RNA (Supplementary Figure 6H). Moreover, the differential expression of the other nine genes monitored by nuclear RNA coincided with total RNA. However, even under these circumstances, more pronounced changes were obtained from nuclear RNA (Figures 6F,G), shown as *ACS1A* and *ACO6.* Notably, the upregulation of the rate-limiting enzyme gene *ACO6* in the synthetic pathway evaluated by nuclear RNA was three times higher than the total RNA.

Taken together, 16/17 genes showed a great difference in the relative expression between nuclear RNA and total RNA, indicating the RNA from the nucleus purified by gwINTACT could reflect the change of the differential gene expression more truly with ethylene treatment. These results laid a foundation for the following study of global gene expression by using RAN-seq and other methods.

## Discussion

In recent years, INTACT has become a popular approach for nuclear purification to measure gene expression and chromatin profiles, which were first described in the *Arabidopsis* root epidermis (Deal and Henikoff, 2010). The original INTACT relied on the WPP-GFP-BLRP nuclear targeting fusion protein co-expressing with the *E. coli* biotin ligase BirA to produce biotinylated nuclei. These biotin-labeled nuclei can be grasped from the total extract pool because of the interaction between streptavidin and biotin (Deal and Henikoff, 2010, 2011). The purified target nucleus from INTACT (with a high yield) has decreased organelle contamination and provides enough pure material, enabling the investigations of rapid changes in gene transcriptional expression (Wang and Deal, 2015; Serafin et al., 2019; You et al., 2019). Because of its superiority, INTACT was applied to the study of other plant species, such as tomato (Ron et al., 2014) and rice (Reynoso et al., 2018). In addition, it has also been used in *Drosophila melanogaster* (Henry et al., 2012; Steiner et al., 2012; Agrawal et al., 2019) and *Xenopus* (Amin et al., 2014) research.

There have been multiple modifications to INTACT that simplified the operation process and enhanced the nuclear purification effect. A magnetic frame was utilized instead of a traditional flow adsorption device to facilitate magnetic bead adsorption (Wang and Deal, 2015), and this considerably shortened the time and eased the experimental operations. Optimization of the outer nuclear envelope protein WPP in the NTF of INTACT has also been studied in plants and animals. There was a coil-coiled domain of a WIP which interacts with the WPP domain (Reynoso et al., 2018). The WIP could anchor to the nuclear envelope by itself and interact with the inner nuclear membrane (Rose and Meier, 2001; Xu et al., 2007). Because of the characteristics of WIP, it was substituted for WPP and was verified to have a more integral association with the nuclear membrane in the previous study of *Oryza* (Rose and Meier, 2001; Xu et al., 2007; Zhao et al., 2008; Reynoso et al., 2018). For the research on *Drosophila* (Henry et al., 2012), the WPP domain was replaced by a UNC-84 protein from *C. elegans* embedded in the inner nuclear membrane of all eukaryotes, and the target nucleus was recovered with 99% purity. Furthermore, the BLRP, which served as a substrate for the BirA in traditional INTACT, was replaced with GFP to label the nucleus. The GFP-labeled nuclei were then captured by the GFP antibody-coupled magnetic beads, which improved the purity of the *Arabidopsis* embryo nucleus between 70 and 90% (Park et al., 2016). Seeking a more convenient INTACT strategy with better performance on the nucleus purification, we integrated the two aspects of the previous INTACT alterations. One uses the C-terminal region of WIP (as an alternative to the WPP domain) for a more stable and robust combination with the outer nucleus envelope (Reynoso et al., 2018). The other one chooses enhanced GFP to label the nucleus and observe fluorescence (Henry et al., 2012; Park et al., 2016). In addition, instead of the commonly used CaMV 35S promoter, the retooled nucleus targeting fusion (NTF) protein was driven by mas promoter, which was proved to enhance the performance of the editing events by driving Cas9 in the previous study (An et al., 2021). Finally, the new *Sl*NTF based on mas-GW was obtained for subsequent evaluation (Figures 1, 2).

With the progress of INTACT, the application of this technology in plants is not only limited to *Arabidopsis*, rice, and maize but also to fleshy tomatoes which is one of the most extensively cultivated and economically essential crops worldwide (Zhao et al., 2021). However, most applications are still limited in the roots or seedlings. There is less research on the leaf cells, which are critical model tissues for the study of photosynthesis and respiration due to the severe chloroplast contamination. Similarly, fruit tissues are vital materials for quality and nutrition research, facing the same challenge induced by the rare nuclei. Our study aimed to provide the INTACT technique in various tissues of tomatoes (*S. lycopersicum*) with excellent purification outcomes. Therefore, two stably inherited *Sl*NTFs overexpressed tomato lines were obtained (GW2 and BGW), with proper nuclear envelope anchoring (Figure 3) and an intensive purification effect (Figure 4 and Supplementary Figures 2, 3). Specifically, the chloroplast removal rate was above 95% in the leaf tissues. The nuclear yield in flower and fruit tissues was doubled to the traditional INTACT method. Thus, this study first introduced the gwINTACT in various tomato tissues for nuclear isolation and purification, providing critical materials for accurately deciphering gene transcriptional expression in tomatoes.

There are many studies on tomato gene transcriptional expression during the growth and fruit ripening. Most studies are based on the total RNA, which only indicate the intracellular homeostasis RNA (Park et al., 2012). In this study, leaves from GW2 lines were treated with ethylene. The discrepancies in the relative expression change of 17 ethylene-related genes were detected by total RNA and nuclear RNA (Figure 6 and Supplementary Figure 6). These changes include complete opposite (Supplementary Figures 6B,D,E,O), changes only in one kind of RNA (Supplementary Figures 6A,H,K,N), and more pronounced changes (Supplementary Figures 6C,F,G,I,J,L,M,P,Q). Many of these essential genes have previously been studied in *Arabidopsis* and tomatoes. *ACS6*, encoding a crucial ACC synthase in the ethylene biosynthesis pathway, was verified to be induced by ethylene in *Arabidopsis* leaves (Arteca and Arteca, 1999). The levels of *ACS6* mRNA transcripts will be increased by touch stimulation of tomato seedlings and fruits (Tatsuki and Mori, 1999), and this is consistent with the upregulation of *ACS6* expression in ethylene-treated leaves obtained using nuclear RNA in our study. In contrast, the downregulation of *ACS6* gene expression was detected using total RNA. EIN3/EILs (ethylene insensitive3/EIN3-LIKES) is the primary ethylene response factor in the downstream ethylene signal transduction pathway. The accumulation and stability of EIN3/EILs proteins are mediated by EBF1/EBF2, the F-box protein family (An et al., 2010). *SlEBF1* showed a clear responsiveness to ethylene with a 4-fold increase in ethylene-treated tomato seedlings in the previous study (Yang et al., 2009), which is corresponded with our results of upregulated *EBF1* expression in nuclear RNA and total RNA.

In this regard, based on the nuclear RNA from gwINTACT, we detected the relative expression levels of 17 essential genes involved in the ethylene synthesis and signal transduction pathways in leaves and accurately assessed the regulation of ethylene based on its expression. The gwINTACT laid a foundation for the more extensive application of INTACT to the kinds of plant tissues involved in the plant growth and development, as well as fruit ripening. In addition, gwINTACT could be combined with the ChIP-seq or ATAC-seq (Assay for Transposase Accessible Chromatin using sequencing) for a comprehensive open chromatin study to conduct general transcriptional regulation analysis. These results facilitated the further research on the regulatory network of tomato fruit ripening and provided ideas and references for research on other important plant species.

## Data Availability Statement

The original contributions presented in the study are publicly available. These data can be found here: National Center for Biotechnology Information (NCBI) BioProject database under accession number PRJNA795585 (https://www.ncbi.nlm.nih.gov/bioproject/PRJNA795585).

## Author Contributions

YC, JG, and BZ designed and led the project. YC, JG, YD, and LM performed the experiments. BZ, DF, HZ, and GQ supervised the experiments. YC analyzed the data and wrote the manuscript. BZ, YL, and PW edited the manuscript. All authors contributed to the article and approved the submitted version.

## Conflict of Interest

The authors declare that the research was conducted in the absence of any commercial or financial relationships that could be construed as a potential conflict of interest.

## Publisher’s Note

All claims expressed in this article are solely those of the authors and do not necessarily represent those of their affiliated organizations, or those of the publisher, the editors and the reviewers. Any product that may be evaluated in this article, or claim that may be made by its manufacturer, is not guaranteed or endorsed by the publisher.

## Footnotes

1
https://www.ncbi.nlm.nih.gov/

2
https://services.healthtech.dtu.dk/service.php?DeepLoc-1.0

3
http://cello.life.nctu.edu.tw/cello2go/

## Abbreviations

GW: eGFP-*Sl*WIP2
GW1: pCAMBIA-35S-eGFP-*Sl*WIP2
GW2: pCAMBIA-mas-eGFP-*Sl*WIP2
BGW: pCAMBIA-35S-BirA-nos-mas-BLRP-eGFP-*Sl*WIP2.

## References

[B1] Agrawal P. Chung P. Heberlein U. Kent C. (2019). Enabling cell-type-specific behavioral epigenetics in Drosophila: a modified high-yield INTACT method reveals the impact of social environment on the epigenetic landscape in dopaminergic neurons. *BMC Biol.* 17:30. 10.1186/s12915-019-0646-4 30967153 PMC6456965

[B2] Amin N. M. Greco T. M. Kuchenbrod L. M. Rigney M. M. Chung M. I. Wallingford J. B. (2014). Proteomic profiling of cardiac tissue by isolation of nuclei tagged in specific cell types (INTACT). *Development* 141 962–973. 10.1242/dev.098327 24496632 PMC3912835

[B3] An F. Zhao Q. Ji Y. Li W. Jiang Z. Yu X. (2010). Ethylene-induced stabilization of ETHYLENE INSENSITIVE3 and EIN3-LIKE1 is mediated by proteasomal degradation of EIN3 binding F-box 1 and 2 that requires EIN2 in Arabidopsis. *Plant Cell* 22 2384–2401. 10.1105/tpc.110.076588 20647342 PMC2929093

[B4] An Y. Geng Y. Yao J. Wang C. Du J. (2021). An Improved CRISPR/Cas9 System for Genome Editing in Populus by Using Mannopine Synthase (MAS) Promoter. *Front. Plant Sci.* 12:703546. 10.3389/fpls.2021.703546 34322148 PMC8311491

[B5] Arteca J. M. Arteca R. N. (1999). A multi-responsive gene encoding 1-aminocyclopropane-1-carboxylate synthase (ACS6) in mature Arabidopsis leaves. *Plant Mol. Biol.* 39 209–219. 10.1023/a:1006177902093 10080689

[B6] Deal R. B. Henikoff S. (2010). A simple method for gene expression and chromatin profiling of individual cell types within a tissue. *Dev. Cell* 18 1030–1040. 10.1016/j.devcel.2010.05.013 20627084 PMC2905389

[B7] Deal R. B. Henikoff S. (2011). The INTACT method for cell type-specific gene expression and chromatin profiling in Arabidopsis thaliana. *Nat. Protoc.* 6 56–68. 10.1038/nprot.2010.175 21212783 PMC7219316

[B8] Del Toro-De Leon G. Kohler C. (2019). Endosperm-specific transcriptome analysis by applying the INTACT system. *Plant Reprod.* 32 55–61. 10.1007/s00497-018-00356-3 30588542

[B9] Entcheva P. Phillips D. A. Streit W. R. (2002). Functional analysis of Sinorhizobium meliloti genes involved in biotin synthesis and transport. *Appl. Environ. Microbiol.* 68 2843–2848. 10.1128/aem.68.6.2843-2848.2002 12039741 PMC123963

[B10] Fazal F. M. Han S. Parker K. R. Kaewsapsak P. Xu J. Boettiger A. N. (2019). Atlas of Subcellular RNA Localization Revealed by APEX-Seq. *Cell* 178 473–490.e26. 10.1016/j.cell.2019.05.027 31230715 PMC6786773

[B11] Giovannoni J. J. (2007). Fruit ripening mutants yield insights into ripening control. *Curr. Opin. Plant Biol.* 10 283–289. 10.1016/j.pbi.2007.04.008 17442612

[B12] Henry G. L. Davis F. P. Picard S. Eddy S. R. (2012). Cell type-specific genomics of Drosophila neurons. *Nucleic Acids Res.* 40 9691–9704. 10.1093/nar/gks671 22855560 PMC3479168

[B13] Ikeda M. Nagashima T. Nakamura E. Kato R. Ohshita M. Hayashi M. (2017). In Vivo Roles of Fatty Acid Biosynthesis Enzymes in Biosynthesis of Biotin and α-Lipoic Acid in Corynebacterium glutamicum. *Appl. Environ. Microbiol.* 83 e01322–17. 10.1128/aem.01322-17 28754705 PMC5601351

[B14] Jacob F. Monod J. (1961). Genetic regulatory mechanisms in the synthesis of proteins. *J. Mol. Biol.* 3 318–356. 10.1016/s0022-2836(61)80072-7 13718526

[B15] Jitrapakdee S. Wallace J. C. (2003). The biotin enzyme family: conserved structural motifs and domain rearrangements. *Curr. Protein Pept. Sci.* 4 217–229. 10.2174/1389203033487199 12769720

[B16] Knowles J. R. (1989). The mechanism of biotin-dependent enzymes. *Annu. Rev. Biochem.* 58 195–221. 10.1146/annurev.bi.58.070189.001211 2673009

[B17] Lau P. P. Xiong W. J. Zhu H. J. Chen S. H. Chan L. (1991). Apolipoprotein B mRNA editing is an intranuclear event that occurs posttranscriptionally coincident with splicing and polyadenylation. *J. Biol. Chem.* 266 20550–20554. 10.1016/s0021-9258(18)54960-7 1939106

[B18] Lei R. Qiao W. Hu F. Jiang H. Zhu S. (2015). A simple and effective method to encapsulate tobacco mesophyll protoplasts to maintain cell viability. *MethodsX* 2 24–32. 10.1016/j.mex.2014.11.004 26150968 PMC4487327

[B19] Li S. Xu H. Ju Z. Cao D. Zhu H. Fu D. (2018). The RIN-MC Fusion of MADS-Box Transcription Factors Has Transcriptional Activity and Modulates Expression of Many Ripening Genes. *Plant Physiol.* 176 891–909. 10.1104/pp.17.01449 29133374 PMC5761797

[B20] Li Z. Guo H. (2018). “Ethylene Treatment in Studying Leaf Senescence in Arabidopsis,” in *Plant Senescence: Methods and Protocols*, ed. Guo Y. (New York, NY: Springer New York), 105–112. 10.1007/978-1-4939-7672-0_8 29392659

[B21] Lin S. Cronan J. E. (2011). Closing in on complete pathways of biotin biosynthesis. *Mol. Biosyst.* 7 1811–1821. 10.1039/c1mb05022b 21437340

[B22] Livak K. J. Schmittgen T. D. (2001). Analysis of relative gene expression data using real-time quantitative PCR and the 2(-Delta Delta C(T)) Method. *Methods* 25 402–408. 10.1006/meth.2001.1262 11846609

[B23] Luo D. L. Ba L. J. Shan W. Kuang J. F. Lu W. J. Chen J. Y. (2017). Involvement of WRKY Transcription Factors in Abscisic-Acid-Induced Cold Tolerance of Banana Fruit. *J. Agric. Food Chem.* 65 3627–3635. 10.1021/acs.jafc.7b00915 28445050

[B24] Nunez E. Fu X. D. Rosenfeld M. G. (2009). Nuclear organization in the 3D space of the nucleus - cause or consequence? *Curr. Opin. Genet. Dev.* 19 424–436. 10.1016/j.gde.2009.07.005 19846290 PMC2796509

[B25] Osorio S. Carneiro R. T. Lytovchenko A. McQuinn R. Sorensen I. Vallarino J. G. (2020). Genetic and metabolic effects of ripening mutations and vine detachment on tomato fruit quality. *Plant Biotechnol. J.* 18 106–118. 10.1111/pbi.13176 31131540 PMC6920187

[B26] Park K. Frost J. M. Adair A. J. Kim D. M. Yun H. Brooks J. S. (2016). Optimized Methods for the Isolation of Arabidopsis Female Central Cells and Their Nuclei. *Mol. Cells* 39 768–775. 10.14348/molcells.2016.0209 27788573 PMC5104886

[B27] Park S. H. Chung P. J. Juntawong P. Bailey-Serres J. Kim Y. S. Jung H. (2012). Posttranscriptional control of photosynthetic mRNA decay under stress conditions requires 3′ and 5′ untranslated regions and correlates with differential polysome association in rice. *Plant Physiol.* 159 1111–1124. 10.1104/pp.112.194928 22566494 PMC3387698

[B28] Peters-Wendisch P. Stansen K. C. Götker S. Wendisch V. F. (2012). Biotin protein ligase from Corynebacterium glutamicum: role for growth and L: -lysine production. *Appl. Microbiol. Biotechnol.* 93 2493–2502. 10.1007/s00253-011-3771-8 22159614

[B29] Piwnicka M. Darzynkiewicz Z. Melamed M. R. (1982). RNA and DNA Content of Isolated Cell Nuclei Measured by Multiparameter Flow Cytometry’. *Cytometry* 3 269–275. 10.1002/cyto.990030407 6185286

[B30] Reynoso M. A. Pauluzzi G. C. Kajala K. Cabanlit S. Velasco J. Bazin J. (2018). Nuclear Transcriptomes at High Resolution Using Retooled INTACT. *Plant Physiol.* 176 270–281. 10.1104/pp.17.00688 28956755 PMC5761756

[B31] Ron M. Kajala K. Pauluzzi G. Wang D. Reynoso M. A. Zumstein K. (2014). Hairy root transformation using Agrobacterium rhizogenes as a tool for exploring cell type-specific gene expression and function using tomato as a model. *Plant Physiol.* 166 455–469. 10.1104/pp.114.239392 24868032 PMC4213079

[B32] Rose A. Meier I. (2001). A domain unique to plant RanGAP is responsible for its targeting to the plant nuclear rim. *Proc. Natl. Acad. Sci. U. S. A.* 98 15377–15382. 10.1073/pnas.261459698 11752475 PMC65037

[B33] Serafin E. K. Chamessian A. Li J. Zhang X. McGann A. Brewer C. L. (2019). Transcriptional profile of spinal dynorphin-lineage interneurons in the developing mouse. *Pain* 160 2380–2397. 10.1097/j.pain.0000000000001636 31166300 PMC7451400

[B34] Steiner F. A. Henikoff S. (2015). Cell type-specific affinity purification of nuclei for chromatin profiling in whole animals. *Methods Mol. Biol.* 1228 3–14. 10.1007/978-1-4939-1680-1_1 25311117

[B35] Steiner F. A. Talbert P. B. Kasinathan S. Deal R. B. Henikoff S. (2012). Cell-type-specific nuclei purification from whole animals for genome-wide expression and chromatin profiling. *Genome Res.* 22 766–777. 10.1101/gr.131748.111 22219512 PMC3317158

[B36] Tatsuki M. Mori H. (1999). Rapid and transient expression of 1-aminocyclopropane-1-carboxylate synthase isogenes by touch and wound stimuli in tomato. *Plant Cell Physiol.* 40 709–715. 10.1093/oxfordjournals.pcp.a029597 10501031

[B37] Tomato Genome C. (2012). The tomato genome sequence provides insights into fleshy fruit evolution. *Nature* 485 635–641. 10.1038/nature11119 22660326 PMC3378239

[B38] Wang D. Deal R. B. (2015). Epigenome profiling of specific plant cell types using a streamlined INTACT protocol and ChIP-seq. *Methods Mol. Biol.* 1284 3–25. 10.1007/978-1-4939-2444-8_1 25757765

[B39] Wang W. Vignani R. Scali M. Cresti M. (2006). A universal and rapid protocol for protein extraction from recalcitrant plant tissues for proteomic analysis. *Electrophoresis* 27 2782–2786. 10.1002/elps.200500722 16732618

[B40] Wang Y. Yuan J. Yang W. Zhu L. Su C. Wang X. (2017). Genome Wide Identification and Expression Profiling of Ethylene Receptor Genes during Soybean Nodulation. *Front. Plant Sci.* 8:859. 10.3389/fpls.2017.00859 28659933 PMC5469071

[B41] Xu X. M. Meulia T. Meier I. (2007). Anchorage of plant RanGAP to the nuclear envelope involves novel nuclear-pore-associated proteins. *Curr. Biol.* 17 1157–1163. 10.1016/j.cub.2007.05.076 17600715

[B42] Yang Y. Wu Y. Pirrello J. Regad F. Bouzayen M. Deng W. (2009). Silencing Sl-EBF1 and Sl-EBF2 expression causes constitutive ethylene response phenotype, accelerated plant senescence, and fruit ripening in tomato. *J. Exp. Bot.* 61 697–708. 10.1093/jxb/erp332 19903730

[B43] You Y. Sawikowska A. Lee J. E. Benstein R. M. Neumann M. Krajewski P. (2019). Phloem Companion Cell-Specific Transcriptomic and Epigenomic Analyses Identify MRF1, a Regulator of Flowering. *Plant Cell* 31 325–345. 10.1105/tpc.17.00714 30670485 PMC6447005

[B44] Zhang C. Barthelson R. A. Lambert G. M. Galbraith D. W. (2008). Global characterization of cell-specific gene expression through fluorescence-activated sorting of nuclei. *Plant Physiol.* 147 30–40. 10.1104/pp.107.115246 18354040 PMC2330299

[B45] Zhao Q. Brkljacic J. Meier I. (2008). Two distinct interacting classes of nuclear envelope-associated coiled-coil proteins are required for the tissue-specific nuclear envelope targeting of Arabidopsis RanGAP. *Plant Cell* 20 1639–1651. 10.1105/tpc.108.059220 18591351 PMC2483365

[B46] Zhao T. Wu T. Pei T. Wang Z. Yang H. Jiang J. (2021). Overexpression of SlGATA17 Promotes Drought Tolerance in Transgenic Tomato Plants by Enhancing Activation of the Phenylpropanoid Biosynthetic Pathway. *Front. Plant Sci.* 12:634888. 10.3389/fpls.2021.634888 33796125 PMC8008128

