# Supplementary material for: Deciphering Precise Gene Transcriptional Expression Using gwINTACT in Tomato
Source: Front Plant Sci. 2022 Apr 14;13:852206. doi: 10.3389/fpls.2022.852206 (PMC9048029; doi:10.3389/fpls.2022.852206)
Supplement: Supplementary file 1 [file Data_Sheet_1.zip › Supplementary_Material/Supplentary Figures.docx]

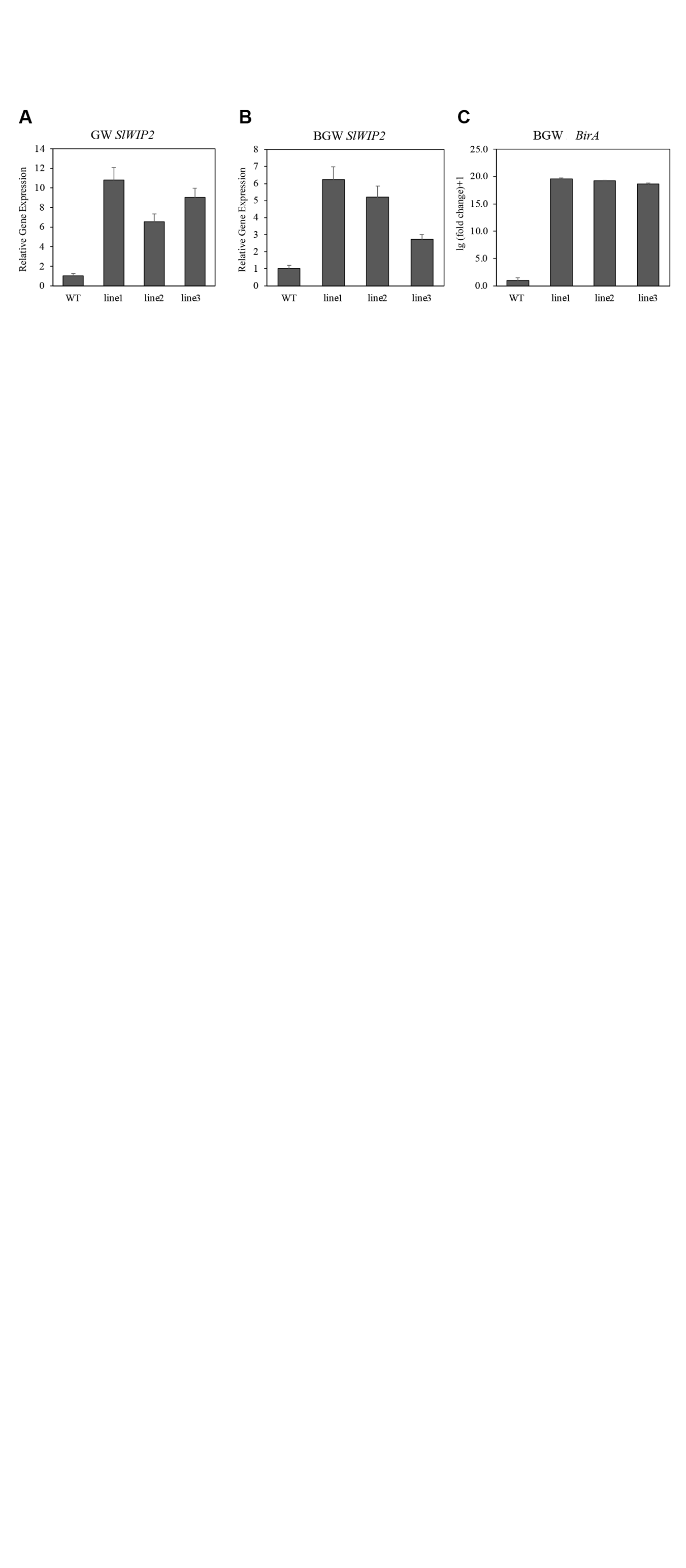


**Supplementary Figure S1. The relative expression of *SlWIP2* and *BirA* in leaves of different NTF lines by RT-qPCR.** Relative expression of *SlWIP2* **(A)**in GW2 lines and *SlWIP2* **(B)***,* *BirA* **(C)** in BGW lines were detected, and WT was used as control plants.


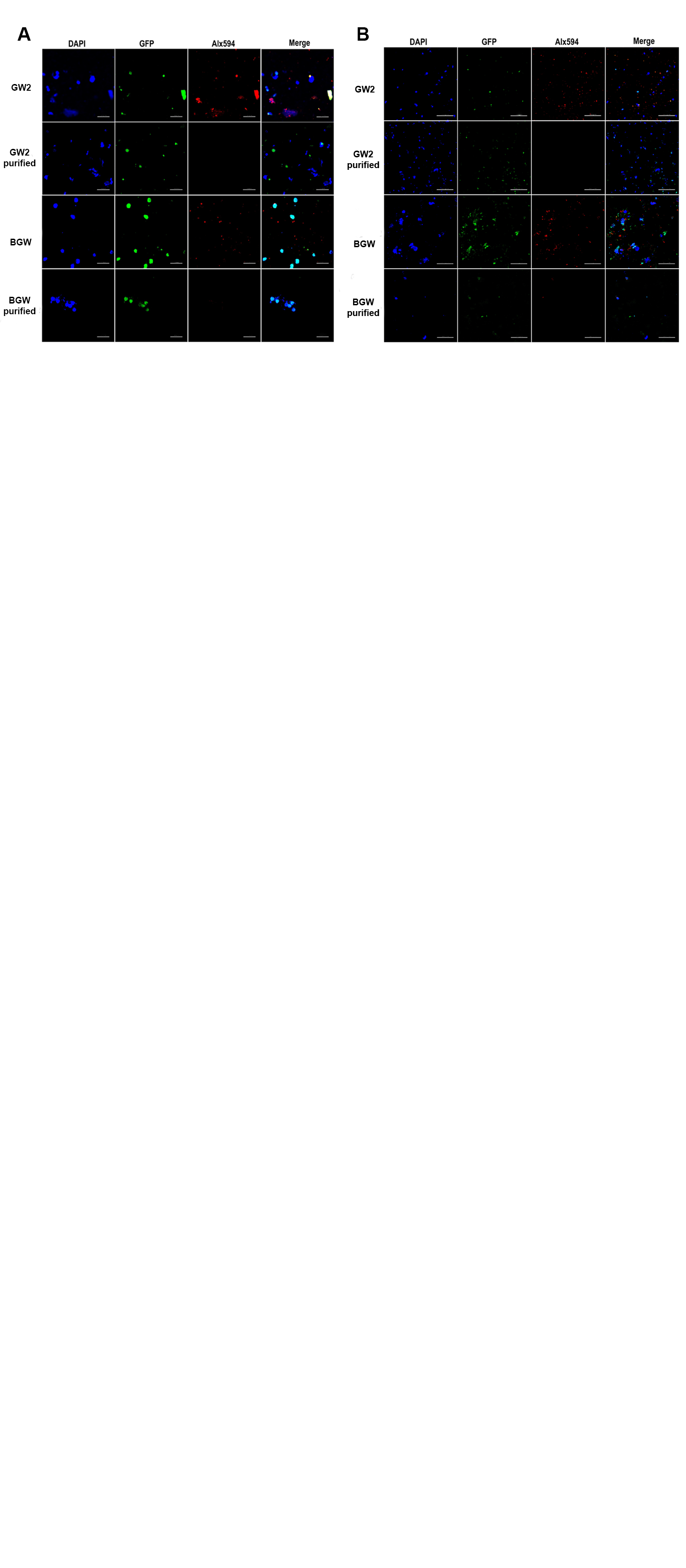


**Supplementary Figure S2. Fluorescence images of nuclei and chloroplasts from different tomato tissues in GW2 lines and BGW lines with purification.** Nuclei were isolated from the flower **(A)** and fruit **(B)** tissues. Nuclei were visualized with DAPI (blue) and GFP (green), chloroplasts were observed using Alx594 (red), and the merged results were shown. Scale bars: 100 µm.


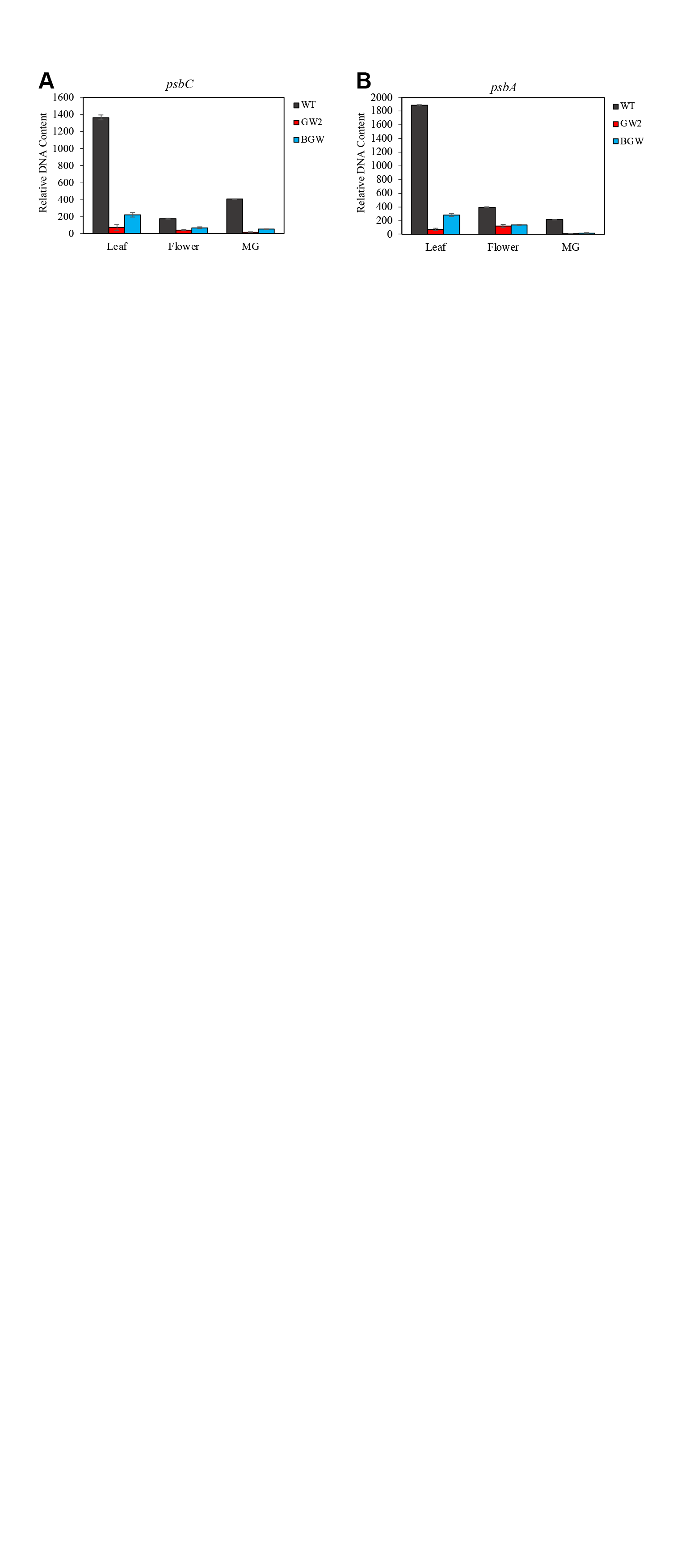


**Supplementary Figure S3. Relative DNA content of chloroplast genes after nuclear purification in different tomato tissues from GW2 lines or BGW lines by PCR.** Two chloroplast-specific genes, *psbC* **(A)** and *psbA* **(B),** were used to represent the relative DNA content of chloroplasts. The amount of reduction compared with WT can reflects the effect of nuclear purification. WT was used as a control group and referred to the chloroplast DNA content of the crude nucleus. Chloroplast removal rate = 1 - relative gene content of chloroplast with purification / relative gene content of chloroplast without purification *100%


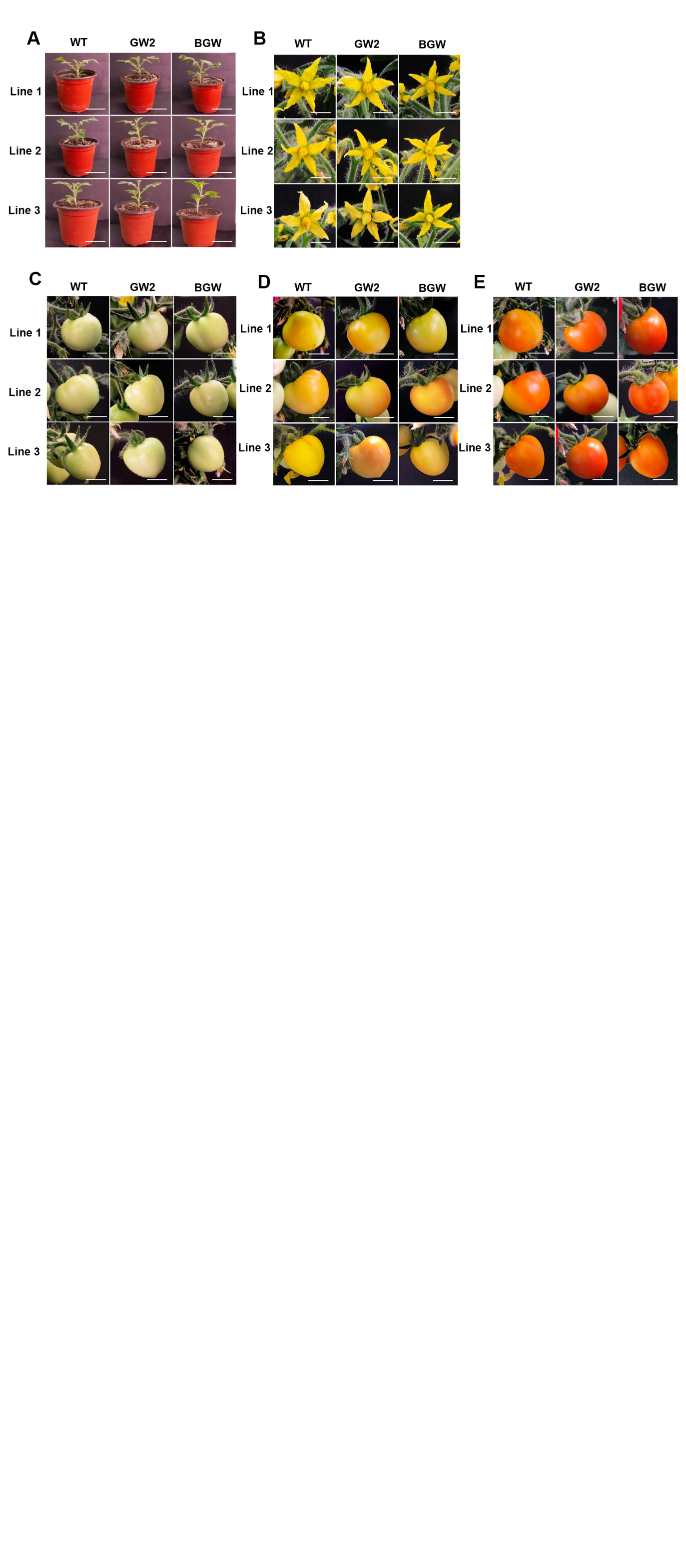


**Supplementary Figure S4. The Phenotype of tomatoes expressing different NTF lines during growth and development and fruit ripening. (A)** 14-d-old seedlings, **(B)** Flowers, **(C, D, E)** Fruit at different ripening stages: MG, B+3, B+5. Scale bars: A, 5 cm. B-E, 1 cm


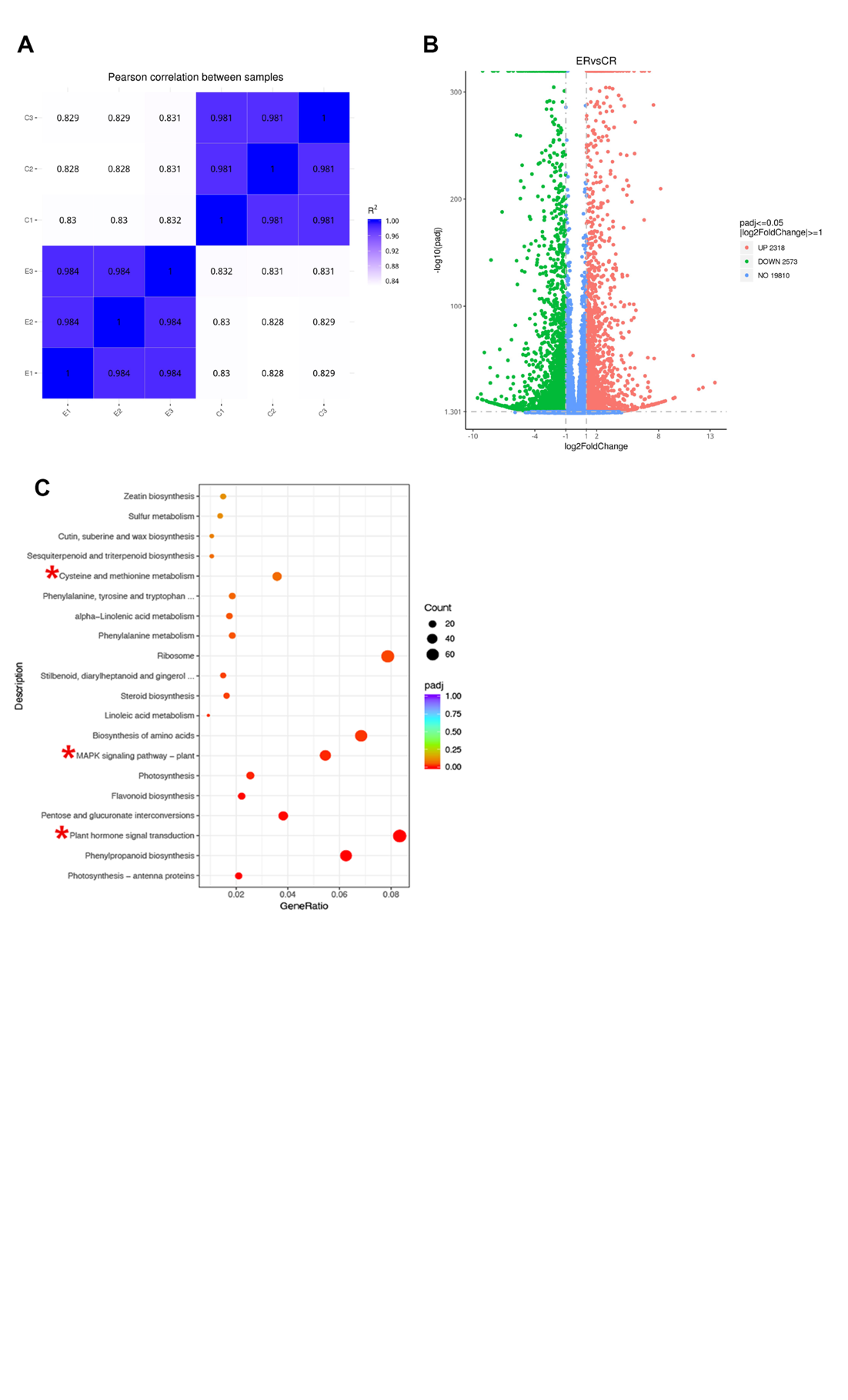


**Supplementary Figure S5. Analysis of differentially expressed genes with ethylene treatment by RNAseq from total RNA of *GW2* tomato leaves. (A)** Pearson correlation coefficient between different sample groups. **(B)** Volcano plot of differentially expressed genes. Green, blue, and red represent genes with up-regulation, down-regulation, and no change, respectively. **(C)** KEGG pathway enrichment.


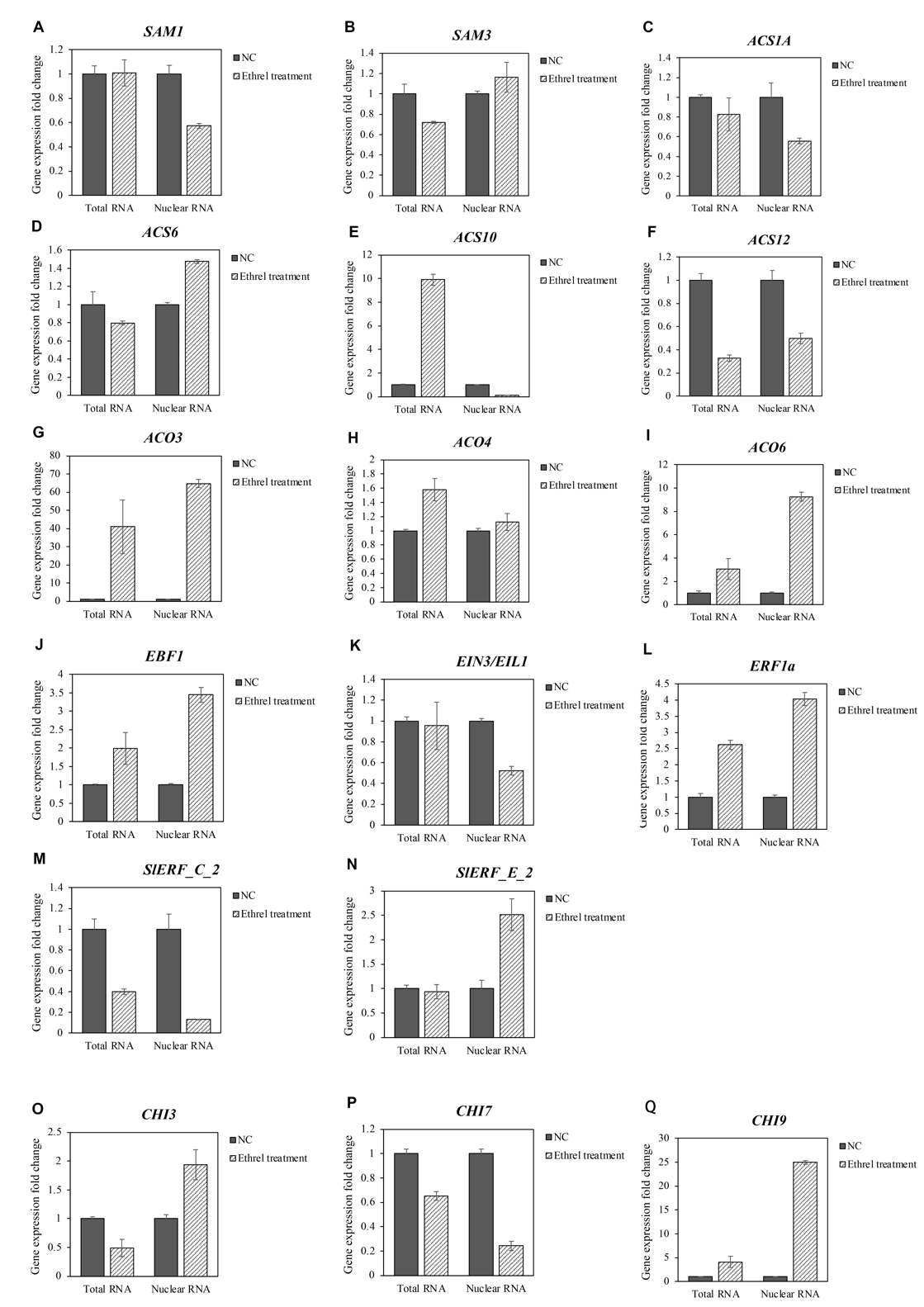


**Supplementary Figure S6. Measurement of relative gene expression fold change with ethylene regulation in total RNA and nuclear RNA of leaves from GW2 lines.** Genes involved in different ethylene metabolism pathways were monitored. **(A-I)** Pathway of ethylene biosynthesis. **(J-N)** Pathway of signal transduction. **(O-Q)** Downstream target genes of ethylene. Differences in the fold change between total RNA and nuclear RNA analysis were notably distinct. Group of GW2 without ethephon treatment is used as a norm control (NC).
